# Relationship of sleep quality, smartphone dependence, and health-related behaviors in female junior college students

**DOI:** 10.1371/journal.pone.0214769

**Published:** 2019-04-03

**Authors:** Po-Yu Wang, Kai-Li Chen, Shang-Yu Yang, Pin-Hsuan Lin

**Affiliations:** 1 Department of Pediatric Emergency, Changhua Christian Children Hospital, Changhua, Taiwan; 2 Department of Nursing, College of Pharmacy and Health Care, Tajen University, Pingtung, Taiwan; 3 Department of Occupational Therapy, College of Medical and Health Science, Asia University, Taichung, Taiwan; 4 Department of Health and Beauty, Shu Zen Junior College of Medicine and Management, Kaohsiung, Taiwan; Graduate School of Medical Science, Kyoto Prefectural University of Medicine, JAPAN

## Abstract

**Introduction:**

Smartphone usage has become commonplace and impact on sleep quality among adolescents. Adolescent girls have a greater tendency toward sleep quality problems. However, relationship of sleep quality, smartphone dependence, and health-related behaviors in female junior college students has not been studied.

**Objectives:**

This study had the two goals: to investigate the relationship between female college students' sleep quality, smartphone dependence, and health-related behaviors, and to identify predictors of sleep quality.

**Methods:**

This study employed a cross-sectional research approach to gather 409 subjects at a junior college in southern Taiwan, and used a structured questionnaire to collect data. The questionnaire consisted of four parts: basic demographic data, the Pittsburgh Sleep Quality Index, assessment of smartphone dependence, and the Health Promoting Lifestyle Profile (HPLP). Logistic regression analysis was employed to check for any association between sleep quality and smartphone dependence or HPLP.

**Results:**

Sleep quality was significantly associated with degree of smartphone dependence, total HPLP score, and scores on the four HPLP subscales of nutritional behavior, self-actualization, interpersonal support, and stress management behavior. The lower the subjects' degree of smartphone dependence was, the better their sleep quality was. Furthermore, the degree of smartphone dependence and total HPLP score were significant predictors of sleep quality.

**Conclusions:**

Smartphone dependence is associated with poor sleep quality among female college students. Improving health-related behaviors (nutritional behavior, self-actualization, interpersonal support, and stress management behavior) can also promote improvement in sleep quality.

## Introduction

Adolescents' sleep quality has been negatively impacted by the use of smartphones [1]. In a study of 309 university students, Demirci, Akgönül [2] suggested that excessive smartphone use may reduce sleep quality. In a study of 2,367 university students, Alosaimi, Alyahya [3] similarly found that excessive use of smartphones may reduce sleep time and be associated with an unhealthy lifestyle (such as lack of exercise). In terms of gender, female university students have a greater tendency to habitual smartphone use than male university students [2, 4], with the consequence that they also have a greater tendency toward sleep problems [5, 6]. The short-wavelength light emitted by smartphone screens at night can interfere with the user’s circadian rhythms, affecting sleep [7]. Although the foregoing studies verified that improper smartphone use may impact sleep quality, they failed to further examine what health-related behaviors can promote better sleep quality.

In Taiwan, approximately 20% of adolescents have sleep quality problems [8]. Sleep quality problems not only negatively impact physiological and psychological health in adolescents [9, 10], but also tend to be accompanied by unhealthy sleep habits, such as the use of cell phones before sleep [11]. Furthermore, adolescents with poor sleep quality also tend to exhibit problematic behavior [12]. In a study of adolescents aged 12–18 years, Min, Kim [13] found that adolescents with poor sleep quality also have a tendency to eat unhealthy foods (such as fast food or instant noodles). In a study of 118 university students, Lai and Say [14] discovered that students with poor sleep quality often have cravings for high-calorie foods. A study of 1,044 university students by Kenney, Lac [15] revealed that students with poor sleep quality have a greater likelihood of alcohol abuse. In addition, the level of sleep quality is also linked to self-harm [16], suicidal tendencies [17], poor mental health and risky health behaviors [18], excessive Internet use [19], and smoking and drinking [20]. Apart from improving physiological and psychological health, excellent sleep quality in adolescents can also boost adolescents' in-school learning performance. In studies of 804 and 165 university students, Baert, Omey [21] and Mirghani, Mohammed [22] respectively found that students with good sleep quality tend to have good academic performance. Good sleep quality may facilitate good school performance in adolescents [23], and may enable them to avoid problems such as obesity [24] and substance abuse [25] as adults.

Research [26] has indicated that sleep quality is linked to health-related behaviors. A healthy lifestyle and good health-related behaviors, such as eating nutritious foods, may be able to improve sleep quality [27]. However, most studies have focused on the negative impact of inappropriate behavior on sleep quality, and devoted little attention to the linkage between different forms of health-promoting behavior (such as exercise and stress management) and sleep quality. In contrast, a systematic investigation of the connection between health-promoting behavior and sleep quality can help teachers and parents instill appropriate health-related behaviors in adolescents, and help adolescents improve their sleep quality.

Although adolescents are in the midst of a physiological and psychological transition to adulthood, the roles they play in their families, schools, and among their peers are very different from those of adults. Because of this difference, it may be inappropriate to rely on the results of studies on adults to assess adolescents' sleep problems. For instance, while adults may encounter sleep problems due to anxiety or depression, adolescents may have sleep problems attributable to a delayed sleep cycle [28]. Furthermore, adolescent sleep patterns may indeed have gender differences, with adolescent girls having a greater tendency toward sleep quality problems than adolescent boys [29, 30]. For instance, female adolescents tend to have more nocturnal awakenings and more nightmares than their male counterparts [31, 32]. Following their first menstruation, adolescent girls have a tendency to suffer from insomnia [33]; an irregular menstrual cycle [34] and menstrual pain [35] will have a negative impact on sleep in adolescent girls. Because of this greater susceptibility to poor sleep quality, an investigation of sleep quality in adolescent girls will help promote good physiological and psychological health as teenage girls transition to adulthood. This study had two goals: to investigate the association between sleep quality, smartphone dependence, and health-related behaviors in female college students; and to find predictors of sleep quality. We hope that the results of this study can lead to a better understanding of what health-promoting activities can improve sleep quality.

## Methods

### Study participants

This study used a cross-sectional research approach to enroll subjects from a junior college in southern Taiwan. Ethical approval for the study was obtained from the National Cheng Kung University Human Research Ethics Committee (No. NCKU HREC-E-106-108-2). In this study, after a research assistant explained the study and obtained participants' written consent, a structured questionnaire was used to collect subject data. The participants could withdraw from the study at any time, and the study did not affect any of their rights and interests. Additionally, written informed consent was obtained from the parents or guardians of the participants who were under the age of 18. Inclusion criteria were (1) female, (2) possession of a smartphone for more than six months, and (3) ability to communicate in Mandarin or the Minnan dialect, and fill out the questionnaire in Chinese. Exclusion criteria were (1) having children, (2) working at night, (3) failure to complete the questionnaire, and (4) presence of mental illness as diagnosed by a physician. Data was collected from September 15, 2017 to November 15, 2017.

### Questionnaire

The questionnaire consisted of four parts. The first part included the basic demographic data of age, body mass index (BMI), smoking habits (smoking at least one cigarette every day during the most recent six months), drinking habits (drinking at least once a week during the most recent six months), and use of a smartphone during the hour immediately before sleep (using a smartphone at least five days each week during the most recent six months).

The second part of the questionnaire assessed sleep quality. This study employed the Chinese version of the Pittsburgh Sleep Quality Index (PSQI). The PSQI was developed by Buysee et al. for use in the clinical assessment of sleep quality, and calls on subjects to record their subjective self-assessment of their sleep quality during the most recent one-month period; it is one of the most commonly used sleep quality assessment scales [36]. The PSQI contains seven assessment indicators; possible scores on each item range from 0 to 3 points, and total scores range from 0 to 21 points. Higher PSQI scores indicate worse sleep quality. The seven indicators on this scale include (1) subjective sleep quality, (2) sleep latent period, (3) sleep time, (4) sleep efficiency, (5) sleep difficulties, (6) daytime functional impairment, and (7) use of sleeping pills. Research has indicated that a total PSQI score of ≤5 indicates good sleep quality, while a score of >5 indicates poor sleep quality [36]. The Chinese version of PSQI was translated by Tsai, Wang [37], who reported that it possesses excellent reliability and validity. In addition, the Cronbach’s α coefficients were 0.73 for PSQI in this study.

The third part of the questionnaire assessed smartphone dependence. Referring to previous studies [38, 39], this part employed four items to gauge the degree of smartphone dependence: (1) I feel uneasy or anxious without a smartphone by my side. (2) I always involuntarily want to stare at my smartphone screen. (3) The first thing I do when I get up in the morning is to check whether there are any calls or text messages on my phone, and check social networking sites, such as Facebook and Line, for messages. (4) I continue to check my smartphone for calls or social networking messages while in class. The questions in this part were scored on a five-point Likert scale (from 1 = do not agree to 5 = completely agree; total score ranging from 4 to 20), with a higher score indicating a greater degree of smartphone dependence. The Cronbach’s α coefficients were 0.76 for smartphone dependence in this study.

The fourth part assessed health-related behaviors, using the Chinese version of the Health Promoting Lifestyle Profile (HPLP), first developed by Walker et al [40]. The HPLP assesses current or recent living habits through six behavioral aspects: (1) nutritional behavior (5 questions), (2) health responsibility behavior (8 questions), (3) self-actualization (8 questions), (4) interpersonal support behavior (6 questions), (5) exercise behavior (4 questions), and (6) stress management behavior (9 questions). All questions are scored on a four-point Likert scale, with scores on each question ranging from 0 to 3 (total score ranging from 0 to 120). Higher scores indicate better health-promoting behavior. As part of the translation process, the number of questions on the scale was reduced to 40 in the Chinese version of HPLP, which was also found to have excellent reliability and validity [41]. The HPLP used in this study had Cronbach's alpha values of 0.95 for the scale as a whole, and 0.80–0.94 for the six behavioral subscales.

### Statistical analysis

This study employed SPSS 22.0 for Mac (IBM Corp., Armonk, NY) for all data analysis. First, descriptive statistics were employed to represent the participants' demographic data, and the subjects were then divided into two groups based on their sleep quality (≤5 indicated good sleep quality and >5 indicated poor sleep quality). Student's t-test and Fisher's exact test were used to determine any significant differences in the demographic data, smartphone dependence, and HPLP scores of the two groups. By using effect size (i.e., Cohen’s *d*; Cohen’s *d* at 0.2 indicates small; 0.5 indicates medium, and 0.8 indicates large [42]), we explored whether the two groups had significantly different performance on the smartphone dependence, and HPLP scores. Pearson’s correlation analysis was employed to examine the association between PSQI total scores and HPLP scores. Logistic regression analysis was employed to check for any association between sleep quality and smartphone dependence or HPLP. Taking sleep quality as the dependent variable of the logistic regression model (0 = poor; 1 = good), we variously used smartphone dependence, the six HPLP aspect scores, and the overall HPLP score as independent variables in the regression model. And with adjusting the model 1 for the variables of age, BMI, smoking habits, drinking habits, and smartphone use before sleep; the model 2 for the model 1 plus the smartphone dependence. Lastly, we used stepwise regression analysis to identify predictors of sleep quality. After putting sleep quality into the stepwise regression model as the dependent variable, we used smartphone dependence, the six HPLP aspect scores, and the overall HPLP score as independent variables in the regression model, and adjusted the model for age, BMI, smoking habits, drinking habits, and smartphone use before sleep.

## Results

After a research assistant explained the study to candidate subjects, 411 female college students agreed to participate and completed the questionnaire. After excluding incomplete questionnaires, a total of 409 valid questionnaires were collected. See Table 1 for basic data on participants. The mean age was 17.35 years (Standard deviation [SD] 1.39, range 15–21 years); 97% of participants had no smoking or drinking habits, and approximately 95% habitually used their smartphones before sleep.

**Table 1:** Demographic, anthropometric, and lifestyle characteristics as well as scores on smartphone dependence and Health Promoting Lifestyle Profile (HPLP).

	Total	Sleep Quality		Effect size	*p*
	N = 409	Poor (n = 213)	Good (n = 196)	Cohen’s *d*	
**Age**	17.35 ± 1.39	17.52 ± 1.45	17.17 ± 1.29	0.26	0.01 ^b^^,^*
**BMI**	20.39 ± 3.55	20.46 ± 3.47	20.31 ± 3.64	0.04	0.67 ^b^
**Smoking habit**					0.06^a^^,^
** No**	398 (97.3%)	204 (95.8%)	194 (99.0%)		
** Yes**	11 (2.7%)	9 (4.2%)	2 (1.0%)		
**Drinking habit**					0.11 ^a^
** No**	399 (97.6%)	205 (96.2%)	194 (99.0%)		
** Yes**	10 (2.4%)	8 (3.8%)	2 (1.0%)		
**Smartphone use before sleep**					0.50 ^a^
** Yes**	388 (94.9%)	204 (95.8%)	184 (93.9%)		
** No**	21 (5.1%)	9 (4.2%)	12 (6.1%)		
**Smartphone dependence score**	12.18 ± 3.35	12.86 ± 3.33	11.44 ± 3.21	0.43	<0.01 ^b^^,^*
**HPLP scores**					
** Nutrition**	8.30 ± 3.07	7.78 ± 2.91	8.87 ± 3.14	0.36	<0.01 ^b^^,^*
** Health responsibility**	7.41 ± 5.33	7.10 ± 5.10	7.75 ± 5.55	0.12	0.22 ^b^
** Self-actualization**	16.19 ± 4.86	15.46 ± 5.01	16.99 ± 4.57	0.32	<0.01 ^b^^,^*
** Interpersonal support**	13.43 ± 3.52	13.04 ± 3.62	13.85 ± 3.37	0.23	0.02 ^b^^,^*
** Exercise**	4.07 ± 3.10	4.01 ± 3.03	4.12 ± 3.17	0.04	0.72 ^b^
** Stress management**	16.86 ± 5.06	16.13 ± 4.88	17.65 ± 5.15	0.30	<0.01 ^b^^,^*
** Total scores**	66.26 ± 18.99	63.52 ± 18.31	69.23 ± 19.32	0.30	<0.01 ^b^^,^*

*p < 0.05;

^a^ Significance of difference determined using Fisher’s exact test;

^b^ Significance of difference determined using Student t-test; BMI: Body mass index

We then analyzed the subjects according to sleep quality. The results of the t-test and Fisher's exact test indicated that subjects with poor sleep quality were older (p<0.05) and had a greater degree of smartphone dependence (p<0.01; small effect size) than subjects with good sleep quality (Table 1). HPLP scores indicated that the nutritional behavior, self-actualization, interpersonal support, and stress management behavior scores of the good sleep quality group were significantly lower than those of the poor sleep quality group (p<0.05–0.01; small effect size); the overall HPLP scores of the good sleep quality group were also significantly higher than those of the poor sleep quality group (p<0.01; small effect size).

The Pearson’s correlation analysis results were shown that the six HPLP aspect scores were all significantly associated with sleep quality (r = -0.13–0.26, p<0.05–0.01), and the overall HPLP score was significantly associated with sleep quality as well (r = -0.25, p<0.01). The logistic regression results are shown in Table 2, which reveals a significant association between smartphone dependence and sleep quality, with a greater the degree of smartphone dependence associated with shorter sleep length (p<0.01). As for HPLP results (model 1 and model 2), nutritional behavior, self-actualization, interpersonal support, stress management behavior, and overall HPLP scores were all significantly associated with sleep quality, with better scores associated with better sleep quality (p<0.05–0.01). Lastly, stepwise regression results revealed that both smartphone dependence and overall HPLP score were significant predictors of sleep quality (B: 0.25, S.E.: 0.05, p<0.01; B:-0.04, S.E.: 0.01, p<0.01).

**Table 2:** Logistic regression analysis for identifying health-related behaviors significantly related to sleep quality.

	OR (95% CI)	*p*
**Smartphone dependence score**	0.88 (0.83–0.94)	<0.01 *
**Model 1**		
**HPLP scores**		
**Nutrition**	1.12 (1.05–1.20)	<0.01 *
**Health responsibility**	1.02 (0.99–1.06)	0.22
**Self-actualization**	1.08 (1.03–1.12)	<0.01 *
**Interpersonal support**	1.08 (1.02–1.14)	0.01*
**Exercise**	1.01 (0.95–1.08)	0.71
**Stress management**	1.07 (1.03–1.11)	<0.01 *
**Total scores**	1.02 (1.01–1.03)	<0.01 *
**Model 2**		
**HPLP scores**		
**Nutrition**	1.11 (1.04–1.19)	<0.01 *
**Health responsibility**	1.02 (0.98–1.06)	0.25
**Self-actualization**	1.08 (1.03–1.13)	<0.01 *
**Interpersonal support**	1.09 (1.02–1.15)	0.01*
**Exercise**	1.01 (0.95–1.08)	0.76
**Stress management**	1.07 (1.02–1.11)	<0.01 *
**Total scores**	1.02 (1.01–1.03)	<0.01 *

Model 1: Adjusted for age, body mass index (BMI), smoking and drinking habits, habitual use of smartphone before sleep.

Model 2: Adjusted for age, body mass index (BMI), smoking and drinking habits, habitual use of smartphone before sleep, and smartphone dependence.

* *p* < 0.05;

HPLP: Health Promoting Lifestyle Profile; OR: Odds ratio; CI: Confidence interval

## Discussion

The findings of this study revealed that the participants' sleep quality was indeed associated with some health-related behaviors, including degree of smartphone dependence, nutritional behavior, self-actualization, interpersonal support, and stress management behavior. Smartphone dependence and overall HPLP score were both predictors of sleep quality. Although smartphone use has now become ubiquitous, and their smart functions make everyday life more convenient in many ways, smartphones also have many negative effects. As the results of this study suggest, smartphone dependence has a significant impact on sleep quality (Tables 1 and 2, regression results). Past studies [2, 4] have already suggested that female college students have a greater tendency than male students toward smartphone dependence or addiction. Unlike male students, who prefer to use their smartphones to play games, most female college students use smartphones to listen to music, view films, and go on social networks [6]. Moreover, long-term use and excessive use of smartphones tends to cause musculoskeletal discomfort [39], a negative mood (anxiety, depression) [2], and difficulty in establishing interpersonal relationships [43]; all of these factors may directly or indirectly affect sleep quality. Furthermore, Table 1 shows that more than 90% of participants use their smartphones immediately before sleep, or view their smartphone screens while trying to sleep (causing exposure to blue light), which may decrease sleep efficiency and increase sleep onset latency [44]. Because of this effect, excessive use of smartphones before sleep may impair the normal sleep process and degrade sleep quality.

This study differed from many earlier studies of sleep quality which generally were limited to investigating the factors which negatively impact sleep quality. In contrast, the investigation of the linkage between sleep quality and health-promoting behavior in this study has helped clarify which health-related behaviors can improve sleep quality among university students, which may therefore suggest feasible actions to mitigate the effect of smartphone use before sleep. The fact that overall HPLP score is a predictor of sleep quality confirms that a healthy lifestyle may have a positive effect on sleep quality. While a healthy lifestyle has always been believed to have a beneficial effect on maintaining or improving health, university students very often have unhealthy lifestyles, such as by getting insufficient sleep, maintaining unhealthy dietary habits, and lacking exercise [45]. Past research [46] has found that over 60% of female college students lack sufficient physical activity, over 40% have unhealthy dietary habits, and approximately 18% have alcohol abuse problems. However, maintaining a healthy lifestyle can help individuals to achieve and maintain good physical and psychological health. For instance, regular exercise can help reduce the feeling of fatigue, reduce anxiety and depression, and thereby improve sleep quality [47].

The findings of this study (Tables 1 and 2) reveal that participants with good nutritional and dietary habits tended to have good sleep quality. Many past studies have shown an association between dietary habits and sleep quality [27]. Such unhealthy dietary habits as not eating breakfast and eating meals at irregular times may cause sleep quality problems [48]. The consumption of carbohydrate-rich foods (such as sweets and noodles) and sugar-sweetened beverages may also have a negative impact on sleep quality [48]. Tanaka, Yatsuya [49] also found an association between protein and carbohydrate intake and poor sleep quality (including difficulty initiating sleep, difficulty maintaining sleep, and poor sleep quality). Maintaining good nutrition and regular dietary habits is consequently very important to sleep quality. In addition, foods that increase the availability of tryptophan, synthetic serotonin, and melatonin are very effective in promoting sleep [50]. Especially in the case of women, maintaining healthy dietary habits (such as a Mediterranean diet) can ease the symptoms of insomnia and enhance sleep quality [51].

The study by Killgore, Kahn-Greene [52] confirms that poor sleep quality is linked to poor intrapersonal functioning (including reduced self-actualization, self-regard, assertiveness, and sense of independence), which implies that a person with poor intrapersonal functioning may also tend to have sleep problems. The findings of the current study indicated that the lower an individual's self-actualization score is, the more likely that person will have poor sleep quality, which is similar to the findings by Killgore, Kahn-Greene [52]. Good self-actualization may facilitate maintaining or raising the individual's level of wellness or self-fulfillment, which can indirectly enhance the individual's psychosocial well-being, positive life expectation and thereby help to boost sleep quality [53]. From the perspective of the HPLP self-actualization subscale items, we recommend that university students cultivate the ability to "appreciate themselves," "value their own achievements," "be aware of their own strengths and weaknesses," and realize that "every day is full of enjoyment and challenges" in order to improve sleep quality.

The findings of this study revealed that participants with poor interpersonal support also tended to have poor sleep quality, which supports the results of Wang, Qin [54] Compared with high school students, university students apparently pay even greater attention to their interpersonal relationships. Since interpersonal support is a basic social need, university students gradually shift their interpersonal support to persons outside their families, while also learning to establish intimate relationships. If interpersonal relationships are handled poorly, this can readily induce physiological and psychological problems [55]. Moreover, interpersonal support also reflects a person's social skills; if an individual has few friends she can rely on, this also implies that she will have difficulty handling tasks involving interpersonal interactions in daily life [56]. Poor intrapersonal functioning or lack of social skills can easily cause poor mood or depression, which will influence sleep [57]. In particular, adolescent women have a greater tendency than adolescent men to encounter social problems, perhaps because women are more likely to respond to social problems with mood changes, while men tend to react with rational thought processes [56, 58].

Many recent studies have verified that the ability to manage stress is linked with sleep quality among university students [27, 59, 60]. In studies of university students, Wallace, Boynton [59] and Lee, Wuertz [60] suggested that stress is a significant predictor of sleep quality, and may lead to insufficient sleep. Lin, Lin [27] found that poor stress management skills are associated with insufficient sleep among university students. Although the current study and previous studies did not directly determine what kinds of stresses will cause university students to have poor sleep quality, the results for the six HPLP domains in this study enable us to infer that some of the stress may derive from intrapersonal functioning and interpersonal support problems. Because of this finding, encouraging students to cultivate self-affirmation, a positive attitude, good interpersonal relationships, and effective stress management techniques will likely help them more effectively improve their sleep quality.

This study has several limitations. First, all sleep and behavioral data in this study was derived from self-reported questionnaire results. Although the scales used in this study (e.g., PSQI) uniformly possess excellent reliability and validity, bias (e.g., recall bias) may have occurred. Second, because all participants in this study attended the same school, our conclusions must remain conservative. Finally, since this was a cross-sectional study, we were only able to determine association between individual variables, and could not infer cause-and-effect relationships. We therefore recommend a follow-up study in the future. Although this study has some limitations, our findings provide empirical evidence of the relationship between sleep quality, smartphone dependence, and health-promoting behaviors, and can suggest effective health-promoting strategies for enhancing sleep quality among female college students.

## Conclusions

Smartphone usage has great impact on sleep quality and health-related behaviors among adolescent girls. The findings of this study revealed that sleep quality is associated with both smartphone dependence and health-related behaviors (including nutritional behavior, self-actualization, interpersonal support, and stress management behavior). In particular, the lower the smartphone dependence and the better the health-related behaviors, the better the individual's sleep quality. Furthermore, degree of smartphone dependence and overall HPLP score were found to be predictors of sleep quality. Apart from shedding light on the effect of smartphone usage on female college students, this study can provide guidance for drafting strategies to promote better sleep quality among female college students.

## Supporting information

S1 Dataset (SAV)
